# Supplementary material for: Estimation of the domestic water demand‒supply scenario and its key driving factors in the Islamabad-Rawalpindi Metropolitan Area, Pakistan
Source: PLoS One. 2025 Mar 10;20(3):e0293927. doi: 10.1371/journal.pone.0293927 (PMC11892837; doi:10.1371/journal.pone.0293927)
Supplement: S1 File — (DOCX) [file pone.0293927.s001.docx]

**ORGANIZATION BASED QUESTIONNAIRE SURVEY ON WATER SCARCITY AND ITS IMPACT ON DEMAND AND SUPPLY IN RAWALPINDI AND ISLAMABAD**

This questionnaire survey is designed for evaluating the scenario of water scarcity in twin cities Islamabad and Rawalpindi for research purpose by a research student of PhD Environmental science. It is hereby to assure that current data will be used only for research purpose and all the information will be kept confidential including your identity. It is requested to kindly take out sometime and answer all the questions accurately. Your cooperation in this regard will be highly acknowledged.

Name of Respondent

Gender

Designation

Institution name

Date

1. What is the growth rate of household with basic water supply in the city?
2. High b) Low c) Average
3. Is there any water harvesting plan available for sustainable water management.
4. Yes b) No
5. If yes, please mention below
6. Does the organization has any rainwater harvesting tanks for water storage?
7. Yes b) No
8. If yes, what is the storage capacity of the tank?

1. Current water abstraction rate is
2. Excessive abstraction b) Within the limit c) Bellow the limit
3. Projected water abstraction rate is
4. Increasing b) Steady c) Decreasing
5. How do you measure the decline in ground water aquifer?
6. Monitoring wells b) Submersible pressure transmitter c) Any other

1. No of tube wells installed in the city.
2. > 200 b) 200- 300 c) < 300
3. What is the production capacity of the tube wells.
4. 18-20 MGD b) < 20 MGD c) > 20 MGD
5. Which are wet months of the year
6. July- august b) September- October c) any other
7. Which are dry months of the year
8. December- January b) February- march c) any other
9. What is the recorded water dearth in last few years?
10. 6 ft b) < 6 c) > 6 ft
11. What is the depth of water aquifer in Rawalpindi Islamabad?
12. 200 ft b) > 200 ft c) < 200 ft
13. How much decline in water depth has been recorded till now.
14. 6 ft in 5 years b) > 6 ft in 5 year c) < 6 ft in 5 years
15. What is per capita domestic water utility?
16. 10 Gallons b) < 10 Gallons c) > 10 Gallons
17. Proportion of population with access to piped supply?
18. > 70% b) Moderate 50 to 70% c) < 50%
19. Do you reuse water in your daily routine?
20. Yes b) No c) Sometimes
21. What is per capita water demand
22. 10 GD b) < 10 GD c) > 10 GD
23. Do you satisfied with the water quality and quantity you receive.
24. Strongly satisfied b) Not satisfied c) Partially satisfied
25. How much water do you use on average per person per day in liter?
26. ˂ 20 b) 20-30 c) 30-50
27. Is there fair distribution of water in your city?
28. Yes b) No
29. How concerned are you about water scarcity in your area.
30. Extremely concerned b) Slightly concerned c) Not concerned at all.
31. Does your organization has waste water treatment plants?
32. Yes b) No c) I don’t know
33. If yes, what is the capacity of Waste Water Treatment Plant (WWTP)?
34. What is the condition of Waste Water Treatment Plants ( WWTP)?
35. Satisfactory b) Non satisfactory c) Average
36. All of the WWTP that were installed are functional till now?
37. Yes b) No c) Some are functional
38. Is there any future plan for increasing decline in ground water aquifer? If yes, please mention
39. What is the per capita water stress index
40. What is water inflow rate to the reservoir?
41. What is outflow rate of water to the city ?
42. What is your role related to water conservation and management?
43. Please mention the production capacity of water supply by surface water resources.
44. Please mention the production capacity of ground water resource.
45. What is collective production capacity of surface and ground water resources?
46. What are the surface water sources of the city?
47. In your opinion what are the reasons of water scarcity that you are facing.
48. In which months do you face severe problem of water scarcity.
49. How is government working on water issues in your area?

Thanks for your time
