# Supplementary material for: Estimation of the domestic water demand‒supply scenario and its key driving factors in the Islamabad-Rawalpindi Metropolitan Area, Pakistan
Source: PLoS One. 2025 Mar 10;20(3):e0293927. doi: 10.1371/journal.pone.0293927 (PMC11892837; doi:10.1371/journal.pone.0293927)
Supplement: Table S2 — (DOCX) [file pone.0293927.s002.docx]

**Table S-2. Demographic Record of Twin cities of Islamabad-Rawalpindi (1951-2017)**

| Years | Population | Urban population | Rural Population | Annual growth rate (%) | Urban  Growth rate (%) | Proportion of urban Population | Population density (person/km^2^) | No of Housing Unit | House Hold Size | Literacy Rate (%) | % distribution | City Ranking |
| --- | --- | --- | --- | --- | --- | --- | --- | --- | --- | --- | --- | --- |
| Islamabad City | | | | | | | | | | | | |
| 1951 | 95,940 | 37,000* | - | - | 2.78 | - | 106 | - | - | - | 0.3 | - |
| 1961 | 117,669 | 47,000* | - | - | 4.44 | - | 130 | - | - | - | 0.3 | - |
| 1972 | 237,549 | 76,641 | 160,908 | 6.20 | 5.48 | 32.26 | 262 | - | - | - | 0.4 | - |
| 1981 | 340,286 | 204,364 | 135922 | 4.49 | 10.05 | 60.06 | 376 |  | 5.2 | 47.8 | 0.4 | - |
| 1998 | 805,235 | 529,180 | 276,055 | 5.19 | 5.19 | 65.72 | 889 | 128753 | 6.2 | 72.88 | 0.6 | 10^th^ |
| 2017 | 2,006,572 | 1,014,825 | 991,747 | 4.90 | 5.15 | 50.60 | 2215 | 170,936 | 6.0 | 81.49 |  | 9^th^ |
| Rawalpindi City | | | | | | | | | | | | |
| 1951 | 2,37,219 | +28.10 | - | - | 3.00 | 28.10 | - | - | - | - | - | 4^th^ |
| 1961 | 340175 | +43.50 | - | - | 4.20 | 43.50 | - | - | - | - | - | 6^th^ |
| 1972 | 614809 | +80.90 | - | - | 5.18 | 80.90 | - | - | - | - | - | 5^th^ |
| 1981 | 794834 | +29.30 | - | - | 3.21 | 29.30 | - | - | - | - | - | 4^th^ |
| 1998 | 1,409,768 | +77.30 | - | - | 2.98 | 77.30 | 1146 | 212,429 | 6.64 | 76.30 | - | 4^th^ |
| 2017 | 2,098,231 | +48.80 | - | - | 6.50 | 48.80 | 8,100 | 470,588 | 5.80 | - | - | 4^th^ |

Source: Federal Bureau of Statistics, PCO, and NIP
