# Supplementary material for: Estimation of the domestic water demand‒supply scenario and its key driving factors in the Islamabad-Rawalpindi Metropolitan Area, Pakistan
Source: PLoS One. 2025 Mar 10;20(3):e0293927. doi: 10.1371/journal.pone.0293927 (PMC11892837; doi:10.1371/journal.pone.0293927)
Supplement: Table S3 — (DOCX) [file pone.0293927.s003.docx]

**Table S-3. Various Types of Housing Units in the Study Area (% age)**

| **Study Area** | **Owned** | **Rented** | **RCC/T-iron**  **ROOF** | **Brick walls** | **One room** |
| --- | --- | --- | --- | --- | --- |
| Islamabad | 37 | 26 | 95 | 99 | 9 |
| Rawalpindi | 51 | 31 | 99 | 100 | 12 |

Source: Pakistan Social and Living Standard Measurement Survey 2014-1
