# Supplementary material for: Estimation of the domestic water demand‒supply scenario and its key driving factors in the Islamabad-Rawalpindi Metropolitan Area, Pakistan
Source: PLoS One. 2025 Mar 10;20(3):e0293927. doi: 10.1371/journal.pone.0293927 (PMC11892837; doi:10.1371/journal.pone.0293927)
Supplement: Table S4 — (DOCX) [file pone.0293927.s004.docx]

**Table S-4. Major Sources of Drinking Water in Households (% age)**

| Sources of Drinking Water | Pakistan* | Punjab* | Islamabad * | Rawalpindi** |
| --- | --- | --- | --- | --- |
| Tap Water | 22 | 13 | 26 | 46.6 |
| Hand Pumps | 23 | 22 | 0 | 0 |
| Motor Pumps | 30 | 42 | 42 | 32.3 |
| Filtration Plants | 10 | 18 | 17 | 13.8 |
| Tankers/Trucks | 4 | 3 | 3 |  |
| Others | - | - | - | 7.4 |

Source: * PSLM, 2019-2020 ** UN-Habitat, 2018
