# Supplementary material for: Estimation of the domestic water demand‒supply scenario and its key driving factors in the Islamabad-Rawalpindi Metropolitan Area, Pakistan
Source: PLoS One. 2025 Mar 10;20(3):e0293927. doi: 10.1371/journal.pone.0293927 (PMC11892837; doi:10.1371/journal.pone.0293927)
Supplement: Table S5 — (DOCX) [file pone.0293927.s005.docx]

**Table S-5. Water demand for a single house having 4-person accommodation in Islamabad City.**

| **Water demand** | **Quantity (liters)** |
| --- | --- |
| CDA Standard | 227 liters/person/day |
| Daily | 908 |
| Monthly | 27,240 |
| Seasonally (6 months) | 163,440 |
| Yearly | 326,880 |

Source: CDA, 2021
