# Supplementary material for: Estimation of the domestic water demand‒supply scenario and its key driving factors in the Islamabad-Rawalpindi Metropolitan Area, Pakistan
Source: PLoS One. 2025 Mar 10;20(3):e0293927. doi: 10.1371/journal.pone.0293927 (PMC11892837; doi:10.1371/journal.pone.0293927)
Supplement: Table S6 — (DOCX) [file pone.0293927.s006.docx]

**Table S-6. Water demand and supply forecast in Islamabad City from 2017-2050.**

| **Total Water Supply and Demand in Three Different Scenarios in Islamabad** | | | | | | | |
| --- | --- | --- | --- | --- | --- | --- | --- |
| **Scenario- A (Business as Usual)** | | | | | | | |
| Year | Population | Per Capita Water Consumption (G/D) | Water Supply (MGD) | Distribution Losses (%) | Availability at user end | Total Water Demand (MGD) | Deficit (MGD) |
| 2017 | 1014825.00 | 60.00 | 65.00 | 35.00 | 42.25 | 60.89 | 18.64 |
| 2020 | 1179828.00 | 60.00 | 65.00 | 35.00 | 42.25 | 70.79 | 28.54 |
| 2025 | 1516580.17 | 60.00 | 65.00 | 35.00 | 42.25 | 90.99 | 48.74 |
| 2030 | 1949448.46 | 60.00 | 65.00 | 35.00 | 42.25 | 116.97 | 74.72 |
| 2035 | 2505867.73 | 60.00 | 65.00 | 35.00 | 42.25 | 150.35 | 108.10 |
| 2040 | 3221102.38 | 60.00 | 65.00 | 35.00 | 42.25 | 193.27 | 151.02 |
| 2045 | 4140482.12 | 60.00 | 65.00 | 35.00 | 42.25 | 248.43 | 206.18 |
| 2050 | 5322274.85 | 60.00 | 65.00 | 35.00 | 42.25 | 319.34 | 277.09 |
|  |  |  |  |  |  |  |  |
| **Scenario-B (Gradual Improvement in Water Losses)** | | | | | | | |
| Year | Population | Per Capita Water Consumption (G/D) | Water Supply (MGD) | Distribution losses (%) | Availability at user end | Total Water Demand (MGD) | Deficit (MGD) |
| 2017 | 1014825.00 | 60.00 | 65.00 | 35.00 | 42.25 | 60.89 | 18.64 |
| 2020 | 1179828.00 | 60.00 | 65.00 | 35.00 | 42.25 | 70.79 | 28.54 |
| 2025 | 1516580.17 | 60.00 | 65.00 | 30.00 | 45.50 | 90.99 | 45.49 |
| 2030 | 1949448.46 | 60.00 | 65.00 | 25.00 | 48.75 | 116.97 | 68.22 |
| 2035 | 2505867.73 | 60.00 | 65.00 | 20.00 | 52.00 | 150.35 | 98.35 |
| 2040 | 3221102.38 | 60.00 | 65.00 | 15.00 | 55.25 | 193.27 | 138.02 |
| 2045 | 4140482.12 | 60.00 | 65.00 | 10.00 | 58.50 | 248.43 | 189.93 |
| 2050 | 5322274.85 | 60.00 | 65.00 | 10.00 | 58.50 | 319.34 | 260.84 |
|  |  |  |  |  |  |  |  |
| **Scenario-C (Without Water Losses)** | | | | | | | |
| Year | Population | Per Capita Water Consumption (G/D) | Water Supply (MGD) | - | - | Total Water Demand (MGD) | Deficit (MGD) |
| 2017 | 1014825.00 | 60.00 | 65.00 | - | - | 60.89 | 4.11 |
| 2020 | 1179828.00 | 60.00 | 65.00 | - | - | 70.79 | 5.79 |
| 2025 | 1516580.17 | 60.00 | 65.00 | - | - | 90.99 | 25.99 |
| 2030 | 1949448.46 | 60.00 | 65.00 | - | - | 116.97 | 51.97 |
| 2035 | 2505867.73 | 60.00 | 65.00 | - | - | 150.35 | 85.35 |
| 2040 | 3221102.38 | 60.00 | 65.00 | - | - | 193.27 | 128.27 |
| 2045 | 4140482.12 | 60.00 | 65.00 | - | - | 248.43 | 183.43 |
| 2050 | 5322274.85 | 60.00 | 65.00 | - | - | 319.34 | 254.34 |
