# Supplementary material for: Estimation of the domestic water demand‒supply scenario and its key driving factors in the Islamabad-Rawalpindi Metropolitan Area, Pakistan
Source: PLoS One. 2025 Mar 10;20(3):e0293927. doi: 10.1371/journal.pone.0293927 (PMC11892837; doi:10.1371/journal.pone.0293927)
Supplement: Table S7 — (DOCX) [file pone.0293927.s007.docx]

**Table S-7. Water demand and supply forecast in Rawalpindi City from 2017-2050.**

| **Total Water Supply and Demand in Three Difference Scenarios in Rawalpindi** | | | | | | | |
| --- | --- | --- | --- | --- | --- | --- | --- |
| **Scenario-A (Business As Usual)** | | | | | | | |
| Year | Population | Per Capita Water Consumption (G/D) | Water Supply (MGD) | Distribution Losses (%) | Availability at user end | Total Water Demand (MGD) | Deficit (MGD) |
| 2017 | 2,098,231 | 35.00 | 54.07 | 35.00 | 35.14 | 73.44 | 38.30 |
| 2020 | 2234527.57 | 35.00 | 54.07 | 35.00 | 35.14 | 78.21 | 43.07 |
| 2025 | 2481645.52 | 35.00 | 54.07 | 35.00 | 35.14 | 86.86 | 51.72 |
| 2030 | 2756092.43 | 35.00 | 54.07 | 35.00 | 35.14 | 96.46 | 61.32 |
| 2035 | 3060890.61 | 35.00 | 54.07 | 35.00 | 35.14 | 107.13 | 71.99 |
| 2040 | 3399396.63 | 35.00 | 54.07 | 35.00 | 35.14 | 118.98 | 83.84 |
| 2045 | 3775338.27 | 35.00 | 54.07 | 35.00 | 35.14 | 132.14 | 97.00 |
| 2050 | 4192855.55 | 35.00 | 54.07 | 35.00 | 35.14 | 146.75 | 111.61 |
|  |  |  |  |  |  |  |  |
| **Scenario-B (Gradual improvement in Water Losses** | | | | | | | |
| Year | Population | Per Capita Water Consumption (G/D) | Water Supply (MGD) | Distribution Losses (%) | Availability at user end | Total Water Demand (MGD) | Deficit (MGD) |
| 2017 | 2,098,231 | 35.00 | 54.07 | 35.00 | 35.14 | 73.44 | 38.30 |
| 2020 | 2234527.57 | 35.00 | 54.07 | 35.00 | 35.14 | 78.21 | 43.07 |
| 2025 | 2481645.52 | 35.00 | 54.07 | 30.00 | 37.85 | 86.86 | 49.01 |
| 2030 | 2756092.43 | 35.00 | 54.07 | 25.00 | 40.55 | 96.46 | 55.91 |
| 2035 | 3060890.61 | 35.00 | 54.07 | 20.00 | 43.26 | 107.13 | 63.87 |
| 2040 | 3399396.63 | 35.00 | 54.07 | 15.00 | 45.96 | 118.98 | 73.02 |
| 2045 | 3775338.27 | 35.00 | 54.07 | 10.00 | 48.66 | 132.14 | 83.48 |
| 2050 | 4192855.55 | 35.00 | 54.07 | 10.00 | 48.66 | 146.75 | 98.09 |
|  | | |  |  |  |  |  |
| **Scenario C (Without water Losses)** | | | | | | | |
| Year | Population | Per Capita Water Consumption (G/D) | Water Supply (MGD) | - | - | Total Water Demand (MGD) | Deficit (MGD) |
| 2017 | 2,098,231 | 35.00 | 54.07 | - | - | 73.44 | 19.37 |
| 2020 | 2234527.57 | 35.00 | 54.07 | - | - | 78.21 | 24.14 |
| 2025 | 2481645.52 | 35.00 | 54.07 | - | - | 86.86 | 32.79 |
| 2030 | 2756092.43 | 35.00 | 54.07 | - | - | 96.46 | 42.39 |
| 2035 | 3060890.61 | 35.00 | 54.07 | - | - | 107.13 | 53.06 |
| 2040 | 3399396.63 | 35.00 | 54.07 | - | - | 118.98 | 64.91 |
| 2045 | 3775338.27 | 35.00 | 54.07 | - | - | 132.14 | 78.07 |
| 2050 | 4192855.55 | 35.00 | 54.07 | - | - | 146.75 | 92.68 |
