# Supplementary material for: Estimation of the domestic water demand‒supply scenario and its key driving factors in the Islamabad-Rawalpindi Metropolitan Area, Pakistan
Source: PLoS One. 2025 Mar 10;20(3):e0293927. doi: 10.1371/journal.pone.0293927 (PMC11892837; doi:10.1371/journal.pone.0293927)
Supplement: Table S8 — (DOCX) [file pone.0293927.s008.docx]

**S-8 Key stakeholders**

Following stakeholders were consulted and incorporating their suggestions and expert opinions while designing, executing and implementing present study.

| **Sr. No** | **Stake holder** | **Area of expertise** |
| --- | --- | --- |
|  | Dr. Muhammad Ashraf | Co-supervisor / Chairman PCRWR |
|  | Dr. Naveed Iqbal Gondal | GIS expert and hydrologist |
|  | Dr. Hifza Rasheed | Director General (WQ) Water quality expert |
|  | Mr. Ibtisam Asmat | Hydrologist , Assistant Director |
|  | Engr. Faizan-ul-Hassan | Director water management (WM) |
|  | Engr. Muhammad Hamza | Hydrologist , Assistant Director |
|  | Ms. Mufeeza Ahsan | Research officer, water quality |
|  | Ms. Saiqa Imran | Senior Research officer, water quality |
