# Supplementary material for: Estimation of the domestic water demand‒supply scenario and its key driving factors in the Islamabad-Rawalpindi Metropolitan Area, Pakistan
Source: PLoS One. 2025 Mar 10;20(3):e0293927. doi: 10.1371/journal.pone.0293927 (PMC11892837; doi:10.1371/journal.pone.0293927)
Supplement: Attachment — Submitted filename: Reviewer 5 - Response file.docx [file pone.0293927.s014.docx]

**Estimation of the domestic water demand‒supply scenario and its key driving factors in the Islamabad-Rawalpindi Metropolitan Area, Pakistan**.

**Manuscript ID:** PONE-D-23-14511

**Reply – Reviewer #5**

Dear Reviewer,

We would like to thank you for your valuable comments about our manuscript titled “**Estimation of the domestic water demand‒supply scenario and its key driving factors in the Islamabad-Rawalpindi Metropolitan Area, Pakistan**.” (ID: PONE-D-23-14511). Those comments are all valuable and very helpful for revising and improving our paper, as well as the important guiding significance to our research. We have studied comments carefully, incorporated all necessary corrections which seemed technically sound, and prepared our arguments for the query as considered pertinent in terms of the difference in opinion on logical grounds. We hope it meets the requirements for your kind approval. All changes in the paper are marked in track change mode in the submitted version of the document. The following are responses to the comments.

**Note:** Prefix “C” means Comment, Prefix “R” means Response

C-1. Statistical Analysis

R-1. Error bars and trend lines have been incorporated in all figures. Needful has been done.

C-2. Stakeholder Consultation

R-2. Thank you so much for suggestion to improve quality of the manuscript. Stakeholder consultation part is incorporated in the methodology section of the manuscript. Detail description of all stakeholders is given in supplementary material S-8.

C-3. All references should be checked as per the Journal format.

R-3. All references have been rechecked as per the Journal format. Needful has been done.

C-4. Transparent discussion

R-4. Following stakeholders were already consulted and incorporating their suggestions and expert opinions while designing, executing and implementing present study. Needful has been done.
