# Supplementary material for: Estimation of the domestic water demand‒supply scenario and its key driving factors in the Islamabad-Rawalpindi Metropolitan Area, Pakistan
Source: PLoS One. 2025 Mar 10;20(3):e0293927. doi: 10.1371/journal.pone.0293927 (PMC11892837; doi:10.1371/journal.pone.0293927)
Supplement: Attachment — Submitted filename: Reviewer 2- Response file (1).docx [file pone.0293927.s010.docx]

**Estimation of domestic water demand-supply scenario and its key driving factors in Islamabad-Rawalpindi Metropolitan Area, Pakistan.**

**Manuscript ID:** PONE-D-23-14511

**Reply – Reviewer #2**

Dear Reviewer,

We would like to thank you for your valuable comments about our manuscript titled “**Estimation of domestic water demand -supply scenario and its key driving factors in Islamabad-Rawalpindi Metropolitan Area, Pakistan**” (ID: PONE-D-23-14511). Those comments are all valuable and very helpful for revising and improving our paper, as well as the important guiding significance to our research. We have studied comments carefully, incorporated all necessary corrections which seemed technically sound, and prepared our arguments for the query as considered pertinent in terms of the difference in opinion on logical grounds. We hope it meets the requirements for your kind approval. All changes in the paper are marked in track change mode in the submitted version of the document. The following are responses to the comments.

**Note:** Prefix “C” means Comment, Prefix “R” means Response

Reviewer #2: Comments to authors

I had the opportunity to review your paper “Estimation of domestic water demand-supply scenario and its key driving factors in Islamabad-Rawalpindi Metropolitan Area, Pakistan.” The objectives of the study were: 1) to assess current and projected water demand and supply scenario in twin cities; 2) to relate demographic and urban trends as well as the density of tube wells with water table decline in twin cities.
It is very interesting work and has some merits. However, I have some comments and questions that the authors should address them clearly and scientifically.

**C1**- The introduction- The instruction is too broad. The authors should review and identify clearly (1) the revolution of domestic water demand supply and (2) the key driving factors of high demand and scarcity of domestic water. Also, the introduction would be more interesting if the authors addressed how water demand and scarcity negatively affected the socioeconomic development of a country, esp. Pakistan?

**R1**. Thank you for the valuable input. Introduction is modified in light of your suggestions.

**C2**- Methods and Materials- The data collection procedure was not clearly described. For example, L152: The authors explained that the data was acquired from various organizations through extensive interviews. If so, the authors should indicate the number of respondents who were interviewed and who they were? What questions were used to interview? These are important!

**R2**. We are grateful for your valuable input. We now tried to elaborate Material and methods section to clearly explain the data sources and data analysis process. Questionnaire is added in the Supplementary material (S1 File).

**C3**- Results: Demographic profile of the study area: What is the main point of this section that the authors want to provide to readers? I think this part should be moved to combine with 2.1.

**R3**. We appreciate the in depth study of the reviewer. However, demographic profile of the study is the prime factor contributing towards urbanization, urban sprawl and water demand and supply imbalances. This factor is discussed in results in detail to correlate it with LULCC resulting in groundwater decline on one side and increasing water demand on the other side. Placing this information in Material and Method section will create a missing link in result section.

**C4**: L233-234: …There are approximately 336,182 and 470,588 234 housing units registered in Islamabad and Rawalpindi, respectively… in what year? The authors should add the year to this growth.

**R4**: This statistical record was obtained from National Housing Census record of 2017. Year is added now. Needful has been done.

**C5**: These two sections—3.3.1 and 3.3.2. are more like literature/a review of existing documents/reports. Likely, 3.4 is more about reviewing documents/reports

**R5**: Section 3.3.1 and 3.3.2 display information about situational analysis of groundwater and surface water resources of study area. This information is compiled from CDA, WASA and RCB through questionnaire based interviews. Without this information discussion about supply demand gap will remain incomplete. However, in revised manuscript we tried our level best to remain precise.

**C7**: I saw only two sentences in L365-366 and L389-390 that the authors likely reported some ideas/findings from the survey? However, it is inconsistent and unclear between what is mentioned in the methodology and these parts. In the methodology, the authors said that they conducted intensive interviews in the methodology, and here in the result, the authors said, “…reported by respondent during field survey.” So what exactly had the author done? Meanwhile, there was no other part of the result section explaining the results/findings from the interview or survey. I doubt it!

**R7**: Thank you for your very pertinent question. We have added the questionnaire we used for interview based survey in the S1 File and revised material and method section accordingly. Questionnaire based interviews were conducted to gather data regarding water sources, types, distribution and quantities from officials of CDA, WASA and RCB. Data was analyzed keeping in view the information extracted from these interviews.

**C8**: Conclusion- L480-484: How did the authors come to that conclusion? There was no clue explaining this concern in the result section. Likely in 4.1. Ways toward secure water future. The authors gave many suggestions, but most are from the authors' ideas/perspectives rather than from this research findings. For example, “(i) …an immediate need for formulation of appropriate groundwater 519 legislation/policy….” There is not a sentence talking about the policy gap or need in the research; how did the authors suggest “an immediate need”? It seems out of what the research is doing. At least the authors discussed somewhere in the research about the policy and law before coming up with this suggestion. Specifically, I saw the terms “water theft, unauthorized connections…” which are things that the authors should suggest some actions be taken in order to improve the water supply or reduce the water deficit.

**R8**: We appreciate your suggestion for improvement of the manuscript. We have thoroughly reviewed these sections and revised considerably in light of your suggestions.

**C9**: More importantly, the authors should discuss and suggest what the authors proposed in Scenarios A, B, and C, which are in the scope of the study. If scenarios B and C are the best, how to implement them?

**R9**- Scenarios B and C are practical short term measures that need to be considered by wter supply schemes. Way forward section provide suggestion to implement this scenario.
