# Supplementary material for: Estimation of the domestic water demand‒supply scenario and its key driving factors in the Islamabad-Rawalpindi Metropolitan Area, Pakistan
Source: PLoS One. 2025 Mar 10;20(3):e0293927. doi: 10.1371/journal.pone.0293927 (PMC11892837; doi:10.1371/journal.pone.0293927)
Supplement: Attachment — Submitted filename: Editor Reply File_PLOS One.docx [file pone.0293927.s012.docx]

**Estimation of domestic water demand -Supply Scenario and its key driving factors in Islamabad-Rawalpindi Metropolitan Area, Pakistan.**

**Manuscript ID:** PONE-D-23-14511

**Reply – Editor Comments**

Dear Editor,

We would like to thank you for your valuable comments about our manuscript titled “**Estimation of domestic water demand-supply scenario and its key driving factors in Islamabad-Rawalpindi Metropolitan Area, Pakistan**” (ID: PONE-D-23-14511). Those comments are all valuable and very helpful for revising and improving our paper, as well as the important guiding significance to our research. We have studied comments carefully, incorporated all necessary corrections which seemed technically sound, and on logical grounds. We hope it meets the requirements for your kind approval. All changes in the paper are marked in track change mode in the submitted version of the document. The following are responses to the comments.

**Note:** Prefix “C” means Comment, Prefix “R” means Response

C-1. Please ensure that your manuscript meets PLOS ONE's style requirements, including those for file naming.

R-1. Needful has been done.

C-2. We note that you have stated that you will provide repository information for your data at acceptance. Should your manuscript be accepted for publication, we will hold it until you provide the relevant accession numbers or DOIs necessary to access your data. If you wish to make changes to your Data Availability statement, please describe these changes in your cover letter and we will update your Data Availability statement to reflect the information you provide.

R-2. All the repository information is provided in supplementary tables and excel sheets.

C-3. We note that Figure 1 in your submission contain map images which may be copyrighted. We require you to either (1) present written permission from the copyright holder to publish these figures specifically under the CC BY 4.0 license, or (2) remove the figures from your submission.

R-3. Figure 1- Study Area Map was developed by the author. Background image layer has been acquired from USGS (freely available), therefore no copyright issue.

C-4. Please include a separate caption for each figure in your manuscript.

R-4. Authors already gave separate caption for each figure in the manuscript. Needful has been done.

C-5. Please include captions for your Supporting Information files at the end of your manuscript, and update any in-text citations to match accordingly.

R-5. Supporting information given is incorporated in in-text citations to match accordingly and also give information at the end of manuscript. Needful has been done.

**Additional Editor Comments:**

C-6. Your present paper is not well presented and you need to add more data and analysis. please read following articles.

- [10.5004/dwt.2020.25119](http://dx.doi.org/10.5004/dwt.2020.25119)
- [10.5004/dwt.2019.24156](http://dx.doi.org/10.5004/dwt.2019.24156)

R-6. Thank you so much for your suggestions to improve quality of manuscript. These articles are already reviewed and one is incorporated. More data analysis has been done to improve manuscript like LULU. However, we have another submitted paper on water quality assessment using drinking and irrigational water quality indexes. Therefore, this aspect cannot be included in this manuscript.
